# The Discrimination Against, Health Status and Wellness of People Who Use Drugs in Italian Services: A Survey

**DOI:** 10.3390/medicina55100662

**Published:** 2019-09-30

**Authors:** Concetta Paola Pelullo, Fabio Curcio, Francesco Auriemma, Giuseppe Cefalo, Antonio Fabozzi, Riccardo Rossiello, Laura Spagnoli, Francesco Attena

**Affiliations:** 1 Department of Experimental Medicine, University of Campania “Luigi Vanvitelli”, Via Luciano Armanni 5, 80138 Naples, Italy; concettapaola.pelullo@unicampania.it (C.P.P.); antoniofabozzi70@gmail.com (A.F.); riccardorossiello@gmail.com (R.R.); laura87sp@gmail.com (L.S.); 2 Servizio per le Dipendenze, DS 25, ASLNA1, Uscita sottopasso G.B. Marino, 80125 Naples, Itlay; curcio.fabione@gmail.com (F.C.); nessundolore@libero.it (F.A.); 3 Servizio per le Dipendenze, DS 29, ASLNA1, via Fontanelle 66, 80136 Naples, Italy; cefalog@gmail.com

**Keywords:** discrimination, health status, people who use drug, stigma, wellness

## Abstract

*Background and Objectives:* The aims of this study are to: describe the sociodemographic characteristics and typology of drug addiction among people who use drugs that attend the Servizio per le Dipendenze (SerD), and evaluate the competence and ability of these rehabilitation services to improve their health status and wellness. *Materials and Methods:* A cross-sectional study was conducted from January to July 2017. Patients attending two selected SerD facilities in the city of Naples, Italy were interviewed with a questionnaire gathering information on sociodemographic data, characteristics of drug addiction, characteristics of enrolment at the SerD, self-reported health status and wellness, and reports of the discrimination suffered. *Results:* Among the 451 people interviewed, 72.3% had started taking drugs by the age of 20, and half of them have used drugs within the last year. 54.5% of responders attended SerD for more than 10 years, and the two main reasons for attendance were to get help and to get methadone. 79.4% were declared to have a good/very good/excellent health status at the time of interviewing. 53.7% reported suffering from discrimination. *Conclusions:* Based on our study, discrimination is higher in participants who attended SerD for more than one year, who were formerly in prison, or who were current drug users.

## 1. Introduction

Drug addiction is a multidimensional phenomenon that involves psychic, physical, and sexual problems, the presence of microbial infections, and environmental, cultural, and relational implications [1]. The addict’s self-image plays a very important role because it influences their self-esteem levels and interactions with others, the probability of being helped and cared for, the risk of not complying to therapy, and the risk of falling into depression [2,3].

Therefore, in order to obtain an effective and lasting treatment, people who use drugs (PWUD) must be accepted in specialized centers while maintaining relationships with their families, work environments, and the social groups to which they belong. Maintaining self-esteem is essential to their motivation for self-care and adherence to therapy [4,5,6,7,8,9].

An obstacle to self-esteem and adherence to therapy of PWUD is their perceptions of stigma and discrimination. Stigma and discrimination are similar terms, the first being a belief and an attitude, the second being an action. Therefore, discrimination is part of the process of stigma.

“Stigma” originated from the Greek word that referred to a type of marking or tattoo that was cut or burned into the skin of criminals, slaves, or traitors in order to visibly identify them as blemished or morally polluted persons [10]. Several types of stigma have been identified [11]. Perceived stigma refers to the shame associated with being a PWUD. Enacted stigma is the direct experience of discrimination and rejection from members of society. Self-stigma is defined as the negative thoughts and diminished self-image resulting from identification with the stigmatized group and anticipation of rejection from other people.

Discrimination can be analyzed at different levels: interpersonal, when it occurs between individuals; institutional, or within institutions such as schools, healthcare, housing, and the workplace; structural, which includes social forces not driven by individuals, such as discriminatory legislation [12].

Data from 2017 indicated an increase in the prevalence of cannabis and a stabilization in synthetic stimulant use in Italy, whereas cocaine use seemed to be declining. Cannabis is used by 20.7% of young adults (those aged 15–34 years) and 10.5% of all adults [13], whereas in Europe only France has a higher consumption of the drug (21.5% of young adults and 11.1% of all adults) [14]. In the Italian prisons about 1/4 of population are PWUD and about 1/3 are imprisoned for related-drug crimes (production, sale, transport, distribution, or acquisition) [15].

The Italian drug treatment system includes two sub-systems consisting of public drug dependency service units (known as Servizio per le Dipendenze or SerD) and social-rehabilitative facilities. SerD provide outpatient treatment and are included in the national health system. Instead, the majority of social-rehabilitative facilities are managed by private organizations. They provide treatment mainly to residential or semi-residential patients, but sometimes also provide outpatient treatment.

The aims of this study are to: (1) describe the sociodemographic characteristics, the typology of drug addiction and the discrimination based on a population of PWUD attending the SerD, (2) evaluate the competence and ability of these rehabilitation services to improve the health status and wellness of PWUD.

## 2. Materials and Methods

### 2.1. Setting

The Italian law 309/90 [16] and its subsequent modifications and integrations regulate the services (including SerD, or Servizi per le Dipendenze) for pathological addictions with the aim of preventing and treating psychoactive substance addictions and related diseases. These services also facilitate reinsertion into society for subjects who have dependency problems. SerD, originally created to treat heroin addiction, now deals with broader needs that require new therapeutic strategies to treat cocaine, alcohol, and Delta 9-tetrahydrocannabinoid addictions, in addition to treating behavioral conditions, such as gambling and sex addiction.

A multidisciplinary support team composed of a psychologist, social worker, physician, and nurse provides assistance to PWUD. The subject’s intervention is usually structured around three closely related phases: the welcome, evaluation, and treatment phases. In regards to the evaluation, the following issues are analyzed: the patient’s motivation for using the service; the patient’s drug-related history; the patient’s personality characteristics and the context in which they live; the patient’s current family, social, work, criminal, and other situations; viable hypotheses for a therapeutic plan; and a timeline of implementation for the therapeutic plan.

### 2.2. Data Collection and Participants

A cross-sectional study was conducted from January to July 2017. To reach the defined sample size, we randomly selected two SerD facilities in the city of Naples, Italy.

All patients who presented for their visit at the two selected SerD in the days of interview and gave written consent to participate were eligible. Patients who refused to participate or who did not collaborate were excluded from the study. Patients were interviewed by four physicians over 1–3 days each week on different days of the week for each SerD and during all hours that the centers were open to the public. The interviewers, independent from the SerD team specialized in Epidemiology and Public Health and therefore were well trained in data collection. All patients were interviewed with a structured questionnaire in a room where privacy could be ensured. Participants were informed that all information collected would be confidential and analyzed as an aggregate. The questionnaire was in paper format and filled in by the interviewers. The participation to the interview was free and the participants were not monetarily compensated.

### 2.3. Sample Size

The sample size was estimated to encompass at least 400 subjects, assuming a 50% rate of expected prevalence of the main outcomes (perceived discrimination, wellness and health status), with a margin of error of 5% and level of significance of 95%.

### 2.4. Questionnaire

The questionnaire included information on sociodemographic data: age (continuous), sex (male, female), nationality (Italian, African, European, or Asian), education (primary school, middle school, high school, or college degree), marital status (unmarried, married, or separated/divorced/widower), employment (unemployed or employed), residence (home, homeless, or drug rehabilitation residence), imprisonment (yes/no); characteristics of drug addiction: age of initial drug use (continuous), reason for initial use (open), last incident of drug use (continuous), drugs used (open); characteristics of enrolment at the SerD: duration of enrolment (continuous), reason for enrolment (open); self-reported health status and wellness; discrimination: perceived discrimination (never, rarely, sometimes, often, or always), type of discrimination (open), perpetrators/place of discrimination (open), fear of being discriminated against (1–10 scale), discrimination concealed from (open), feelings about own addiction (open).

### 2.5. Main Outcomes

Concerning the health status and wellness section, the four questions included in the questionnaire were based on the “SF-36 questionnaire” [17], a widely used instrument to check the health status of the general population. However, two questions were modified to satisfy the scope of the study, changing “compared to one year ago” to “compared to after attending SerD.”

To evaluate experiences of discrimination, interviewers asked, “In your lifetime, have you ever been discriminated against, prevented from doing something, or been hassled or made to feel inferior?” [18].

### 2.6. Statistical Analysis

Descriptive analysis was performed for all variables. Univariate analysis was conducted between the main outcome of “Discrimination” and other independent variables, including sex (male = 0, female = 1), age (≥45 = 0, ≤44 = 1), nationality (others = 0, Italian = 1), marital status (married = 0, others = 1), age of initial drug use (≥19 = 0, ≤18 = 1), duration in years of SerD enrolment (<1 = 0, ≥1 = 1), current health status (good/very good/excellent = 0, poor/very poor = 1), health status before SerD enrolment (no change/worse/much worse = 0, better/much better = 1), current wellness level (good/very good/excellent = 0, poor/very poor = 1), wellness level before SerD enrolment (no change/worse/much worse = 0, better/much better = 1), history of imprisonment (no = 0, yes = 1), and last incident of drug use (now = 0, ≥1 month = 1). Only variables associated with the outcome of *p* ≤ 0.25 were subsequently included in the multivariate logistic regression model, and the adjusted OR (odds ratio) has been calculated. Analyses were carried out using SPSS Version 11.0 statistic software package (IBM Corp, Armonk, NY, USA).

A pilot study was carried out with in sample of 20 PWUD, to evaluate the comprehensibility of the wording of each question.

Research ethics committee approval for this study was obtained from the Ethics Committee of the Second University (now called the University of Campania “Luigi Vanvitelli”) of Naples (n prot 926/2016).

## 3. Results

The total number of people registered in the two SerD amount to 1120. However, according to the rules of the services, those who attended the service at least once in the last six months are considered “active attending” subjects, a group that amounts to 680 persons. During the data collection period, we interviewed 451 subjects (301 at the first SerD and 150 at the second SerD) with 10 subjects refusing to participate. Therefore, the response rate was 97.8%, the prevalence rate respect to the “active attending” was 66.3%, and the prevalence rate respect to the people registered was 40.3%.

Most subjects interviewed were over 35 years in age (82.9%), male (91.5%), Italian (76.3%) and had low education levels (primary and middle school) (69%). About half were or had been married (49.9%) and had sons (50.3%). Few subjects were homeless (4.9%), but more than one third were unemployed, and more than one half had been in prison (Table 1).

Most responders (72.3%) started taking drugs by the age of 20 and most had done so for recreational purposes (81.2%). Half had used drugs in the last year and the other half more than a year ago. The number of current users totalled 24.4%. Of all the responders, 54.5% had attended SerD for more than 10 years, and 23.8% had done so for only 5 years. The two main reasons for frequenting a SerD were getting help and accessing methadone (Table 2).

Table 3 compares single-drug users with multiple-drug users. Overall, heroin represented the most commonly used drug (82.6%) among the sample group, followed by cocaine (72.7%) and cannabis (72.2%). Ketamine tended to be used by single-drug users (38.8%). Cannabis and cocaine were rarely used as single drugs and were widespread in multiple-drug users.

Table 4 provides information about subjects’ health statuses and wellness levels in relation to their attendance at the SerD. 79.4% of responders were declared to have a good/very good/excellent health statuses when interviewed, stating that their health was better/much better than before attending SerD (80.2%). Only 10 responders declared to have worse health statuses after attending SerD. Similarly, 69.4% of participants reported their wellness as being good/very good/excellent during the time of interviewing as well as being better/much better than before attending SerD (65.9%).

Reports of responders declaring discrimination and stigma are detailed in Table 5. Out of all the responders, 46.3% had never suffered from discrimination. Among those who reported facing discrimination, the main forms were marginalization (52.4%) and verbal harassment (46.6%). These incidents of discrimination primarily came from friends (43.8%) and people in their workplaces (21.9%). On a scale of 1–10 determining the fear of being discriminated against, the tendency is towards a low level of fear (1–3 = 48.8%). Regarding stigma, most subjects interviewed tended to hide their drug addictions (67.7%). The most commonly presented feelings towards this condition of drug addiction were guilt (46.1%) and regret (21.0%).

In the univariate analysis, discrimination was weakly associated with Italian nationality, poor health status, poor wellness, more than 1 year of attendance at a SerD, being a former prisoner, and being a current drug user. In multivariate analysis only the last three variables were statistically associated with discrimination (Table 6). Disaggregation between the two SerD centers did not show significant differences for discrimination (Table 6) and for all the other investigated variables (data not reported in Table 6).

## 4. Discussion

To the best of our knowledge, this is the first study conducted in Italy about discrimination, health status, and wellness levels in a sample of patients attending SerD centers.

Some considerations about our results arise from the socioeconomic characteristics of participants, their perceived discrimination, health status and wellness. Most patients attending SerD were male, over the age of 35, with low levels of education, attending SerD for long periods of time, and reporting early onsets of drug use. Therefore, this large subgroup of participants maintains chronic conditions of drug dependence with uncertain probabilities of complete recovery due to primarily going to the center to access methadone or similar compounds. Early onset, coupled with recreational motivations to attend SerD, suggests a low critical attitude of these subjects during the tenuous phases of early addiction. However, sex, age, nationality, and marital status did not affect the perceived discrimination.

In our study, 23.7% of enrolled PWUD were non-native to Italy. This percentage is very high compared to the population of foreign residents in Naples in 2017, which is only 5.7% [19]. However, the latter percentage does not take into account the non-native population who are in the country without residence permits, including those with higher risks of using drugs.

Discrimination against PWUD has been investigated in different ways and in relation to different forms, including types of drugs [18], mental illnesses, [20], depression [21], human rights violations [22], racial conditions [12], and also public points of view [20]. The extent of perceived discrimination is greatly variable across studies, ranging from 16.8% [3] to 95.1% [23], partly because it depends on the extreme heterogeneity of the underlying factors and partly because of the different set of criteria employed during the assessments. In our study the subjects’ experiences of discrimination are not very pronounced; in fact, only 22.2% of responders stated to have suffered from discrimination often/always, and 25.9% had a great fear of being discriminated against. Moreover, discrimination was higher in those who attended SerD for more than one year than discrimination faced by those who had been in prison or were current drug users. In another study conducted in the same geographical area about discrimination in lesbian, gay and bisexual individuals, we found a similar percentage of discrimination (28.6%) [24].

Several studies investigated the stigma and discrimination from multiple sources, such as health institutions, friends, family, and the workplace. Discrimination experienced from healthcare may threaten treatment retention among PWUD [25,26] and it is associated with decreased odds of having a regular physician. Family members are an important source of discrimination among PWUD. It has been reported that 75.2% of people experienced discrimination from family due to drug use [6], and that people were unwilling to have a person with drug addiction marry into their family [20]. Discrimination from work colleagues is associated with stress and decreased well-being among PWUD and may also threaten employment status [27,28]. In the current study, friends were the most important source of discrimination. Less important were colleagues in the workplace, healthcare workers, and family. In all cases, discrimination did not seem to affect their health status and wellness.

Many studies on the experiences of drug users have already ascertained the negative effects of drug use on quality of life in general [29,30,31,32,33,34,35]. We observed that most responders reported having a good/very good/excellent health status (79.4%), while at the same time stating that their health improved (80.2%) after attending SerD. In both cases, wellness showed few negative results, or patients reporting worsened health conditions. However, it is important to point out that these results primarily include people undergoing methadone maintenance treatment, which improves health status and wellness.

Recovery in addiction treatment has gained a remarkable consensus [36,37,38,39,40,41,42]. Recovering from alcohol and substance abuse is a process of changing behaviors, requiring an individual to abstain from these substances to improve their health, wellness, and quality of life [43]. This is also the goal of the health operators at the SerD centers. Each patient is assigned to a team with the objective of achieving abstinence and improved healthand wellness according to a structured intervention. However, the lack of detailed assessments on the treated subjects and the lack of data on former patients no longer enrolled in the service prohibits us from ascertaining the full results of such treatments.

This study does have limitations. First, due to the cross-sectional design, it is impossible to establish the direction of the relationship between discrimination and substance abuse. Several studies examining this relationship concluded that discrimination caused substance use rather than the reverse result [44,45]. The group of 451 responders most likely represented a selected subgroup among the 1120 registered users of the two SerD centers, with an over-representation of the 680 most “active attendees” PWUD. This phenomenon by itself may lead to a selection bias, related to the higher proportion of methadone users in this cohort, typically in need of a frequent medication refill. In all likelihood, these users presented a higher chance of being more stable and of being more satisfied with the health provisions offered. Another important limitation is the reliability of the collected data in part because of the difficulty in recalling distant events and in part as a result of the potential tendency to please the interviewers with more positive feedback.

## 5. Conclusions

In our study, discrimination is higher in participants who attended SerD for more than one year, who were formerly in prison, or who were current drug users. Moreover, the two investigated SerD have a low attendance rate compared to the total number of registered people and were used by PWUD mainly to receive methadone, allowing to maintain an acceptable quality of life. Therefore, our results suggest enhancing the complete recovery of people who require methadone and achieving a more active search for non-attending subjects.

## Figures and Tables

**Table 1:** Sociodemographic characteristics of the study population.

	N	%
**Age (years)**		
≤25	10	2.2
26–35	67	14.9
36–45	185	41.0
≥46	189	41.9
**Sex**		
Male	411	91.5
Female	38	8.5
**Nationality**		
Italian	344	76.3
African	51	11.3
European	46	10.2
Asian	10	2.2
**Education**		
Primary School	89	19.7
Middle School	222	49.3
High School	115	25.5
College Degree	25	5.5
**Marital status**		
Unmarried	226	50.1
Married	130	28.8
Separated/Divorced/Widower	95	21.1
**Employment**		
Unemployed	169	37.5
Employed	282	62.5
**Residence**		
Home	425	94.2
Homeless	22	4.9
Drug Rehabilitation Residence	4	0.9
**Imprisonment**		
No	196	43.5
Yes	255	56.5
**Total**	451	100.0

**Table 2:** Characteristics of drug addiction and enrolment at the Servizio per le Dipendenze (SerD).

		N	%
**Age of initial drug use**			
	≤17 years	162	35.9
	18–20 years	164	36.4
	≥21 years	125	27.7
**Reason for initial drug use**			
	Recreational	366	81.2
	Social Distress	85	18.8
**Last incident of drug use**			
	Current	110	24.4
	1–12 months	112	24.8
	1–5 years	104	23.1
	≥6 years	125	27.7
**Duration of SerD enrolment**			
	<1 year	26	5.8
	1–5 years	81	18.0
	6–10 years	98	21.7
	>10 years	246	54.5
Reason for SerD enrolment *			
	Quit/help	312	69.1
	Methadone	157	34.8
	Other	21	4.6
	Missing	6	1.3
**Total**		451	100.0

* Respondents were given the option to select more than one response.

**Table 3:** Types of drugs used.

Type of Drug	Single-Drug Consumers, *n* = 116		Multiple-Drug Consumers *, *n* = 355		Total *, *n* = 451	
	*n*	%	*n*	%	*n*	%
Heroin	46	39.6	325	97.0	371	82.6
Cocaine	14	12.0	314	93.7	328	72.7
Cannabis	2	1.7	324	96.7	326	72.2
Amphetamine and other	3	2.5	199	59.4	202	44.8
Crack	2	1.7	125	37.3	127	28.1
Ketamine	45	38.8	1	0.3	46	10.2
Other	3	2.6	40	11.9	43	9.5
LSD	1	0.8	15	4.4	16	3.5

* Respondents were given the option to select more than one response.

**Table 4:** Health status and wellness levels before and after attending SerD.

		N	%
**Current health status**			
	Very poor	23	5.1
	Poor	70	15.5
	Good	242	53.7
	Very good	87	19.3
	Excellent	29	6.4
**Health status after attending SerD**			
	Much worse	4	0.9
	Worse	6	1.3
	No change	79	17.5
	Better	117	25.9
	Much better	245	54.4
**Current wellness**			
	Very poor	42	9.3
	Poor	96	21.3
	Good	211	46.8
	Very good	73	16.2
	Excellent	29	6.4
**Wellness after attending SerD**			
	Much worse	5	1.1
	Worse	15	3.3
	No change	134	29.7
	Better	122	27.1
	Much better	175	38.8
**Total**		451	100.0

**Table 5:** Discrimination and stigma due to drug addiction.

	N	%
**Discrimination suffered**	
Never	209	46.3
Rarely/Sometimes	142	31.5
Often/Always	100	22.2
Type of discrimination *^,+^		
In the workplace **	58	23.9
Marginalization	127	52.4
Inequality	48	19.8
Verbal harassment	113	46.6
Physical assault	4	1.6
Perpetrator and place of discrimination *^,+^		
Friends	106	43.8
People in work environment	53	21.9
Other	107	44.2
**Fear of discrimination rating**		
1–3	220	48.8
4–7	114	25.3
8–10	117	25.9
**People who addiction status is concealed from ^+^**		
None	146	32.3
Everybody	133	29.4
Family	74	16.4
Other	123	27.2
**Feelings about the starting point of the addiction ^+^**		
Guilt	208	46.1
Regret	95	21.0
Other	164	36.3

* Question offered only to those who suffered discrimination (*n* = 242). ** Workplace includes: mobbing, lay-off and missed hiring. ^+^ Respondents were given the option to select more than one response.

**Table 6:** Discrimination disaggregated for social characteristics, drug addiction, and health status.

				Discrimination		
		No	Yes	Odds Ratio (95% Confidence Interval)	*p* Crude	*p* Adjusted °
		%				
**Sex**	Male	47.0	53.0			
	Female	42.1	57.9	1.22 (0.62–2.38)	*p* = 0.5665	--
**Age**	≤44	47.1	52.9			
	≥45	45.5	54.5	1.07 (0.74–1.54)	*p* = 0.7362	--
**Nationality**	Other	55.1	44.9			
	Italian	43.6	56.4	1.59 (1.03–2.46)	*p* = 0.0374	0.491
**Marital status**	Married	50.2	49.8			
	Other	42.5	57.5	1.37 (0.94–1.98)	*p* = 0.0995	0.760
**Age of initial drug use**	≥19	51.2	48.8			
	≤18	42.3	57.7	1.43 (0.98–2.08)	*p* = 0.0599	0.374
**Duration of SerD enrolment**	<1 year	58.9	41.1			
	≥1 year	42.4	57.6	1.94 (1.25–3.02)	*p* = 0.0031	0.048
**Current health status**	Good ^*^	48.9	51.1			
	Poor ^**^	36.6	63.4	1.66 (1.04–2.66)	*p* = 0.0347	0.200
**Health status after attending SerD**	Worse ^+^	50.6	49.4			
	Better ^++^	45.3	54.7	1.23 (0.78–1.96)	*p* = 0.3733	--
**Current wellness**	Good ^*^	49.8	50.2			
	Poor ^**^	38.4	61.6	1.59 (1.06–2.40)	*p* = 0.0253	0.278
**Wellness after attending SerD**	Better ^++^	46.5	53.5			
	Worse ^+^	46.1	53.9	1.01 (0.69–1.50)	*p* = 0.9419	--
**Imprisonment**	No	54.1	45.9			
	Yes	40.4	59.6	1.74 (1.19–2.53)	*p* = 0.0040	0.016
**Last incident of drug use**	≥1 month	50.4	49.6			
	Current	33.6	66.4	2.01 (1.28–3.15)	*p* = 0.0023	0.027
**SerD**	Serd2	50	50			
	Serd1	44.5	55.5	1.25	*p* = 0.271	--
